# Hepatic inflammation scores correlate with common carotid intima-media thickness in rats with NAFLD induced by a high-fat diet

**DOI:** 10.1186/1746-6148-10-162

**Published:** 2014-07-16

**Authors:** Jing Wu, Hua Zhang, Hao Zheng, Ying Jiang

**Affiliations:** 1 Department of Pathophysiology, Capital Medical University, 10 You An Men Wai Xi Tou Tiao, Beijing 100069, China; 2 Department of Diagnostic Ultrasound, Xuanwu Hospital, Capital Medical University, Beijing 100053, China; 3 Department of Histology and Embryology, Capital Medical University, Beijing 100069, China

**Keywords:** Non-alcoholic fatty liver disease, Atherosclerosis, Ultrasound, Intima-media thickness, Rat

## Abstract

**Background:**

Non-alcoholic fatty liver disease (NAFLD), an emerging public health problem, may be a highly atherogenic condition. But the relationship between fatty liver diseases and carotid atherosclerosis in small-animal is incompletely understood. The purpose of the present study was to evaluate carotid intima-media thickness (IMT) in NAFLD rats using high-frequency ultrasonic diagnostic equipment, and to ascertain if the degree of hepatic pathological changes was associated with carotid IMT.

**Results:**

Liver injury was induced by a high-fat diet for 8, 12 and 16 weeks, separately, in fifty four SD rats (27 treated, 27 controls). Liver echogenicity and IMT of the carotid and aorta were evaluated and compared to histological findings of them. In comparison with the rats in the control group, fatty liver disease in rats was characterized by homogeneous and diffusely increased echogenicity (bright liver), an increased anteroposterior diameter of the liver and serum biochemical changes. Hepatic histological analyses demonstrated indications of simple steatosis in rats induced by an 8-week high-fat diet, and a high-fat diet for 12 weeks and 16 weeks could induce steatohepatitis (NASH) in rats. The 12- and 16-week groups had a significantly higher inflammation scores than those of the control groups. IMT values for the carotid and aorta were remarkably increased in the NASH groups compared with the control groups (P < 0.05). The end-diastolic velocity and systolic peak velocity of the carotid and aorta in the NASH groups were significantly smaller than those in the control group. A significant correlation between the IMT of the carotid with hepatic inflammation score (r^2^ = 0.598, P = 0.001) and the systolic peak velocity of the carotid (r^2^ = −0.342, P = 0.041) were shown in NAFLD rats.

**Conclusion:**

We demonstrated that ultrasound imaging in the diagnosis of fatty liver disease and early atherosclerosis in rats is feasible and efficient, and that carotid IMT increased significantly in NASH rats but not in simple steatotic rats. A significant correlation between the IMT of the carotid artery with hepatic inflammation score were shown in NAFLD rats. This method for non-invasive diagnosis is especially relevant in the research of the pathogenesis and therapy of NAFLD and atherosclerosis using rodent models.

## Background

Increasingly, non-alcoholic fatty liver disease (NAFLD) is recognized as a public health problem in the medical community [1]. NAFLD represents a spectrum of liver disease from steatosis to nonalcoholic steatohepatitis (NASH) and cirrhosis [2]. NAFLD is frequently associated with visceral obesity, dyslipidaemia, insulin resistance and type-2 diabetes mellitus (T2DM) [3]. NAFLD may represent another component of the metabolic syndrome [3,4], which is a highly atherogenic condition. In particular, prospective studies suggest that NAFLD is linked to increased morbidity and mortality from cardiovascular disease (CVD) [5]. NAFLD could signify a substantial risk of cardiovascular events. Hepatic steatosis probably contributes to the pathogenesis of atherosclerosis [6]. Cross-sectional studies have shown that NAFLD is associated with increased carotid artery intima-media thickness (IMT) [6], which is a marker of early atherosclerosis [7].

As observed in humans, NAFLD is associated strongly with obesity, T2DM, and dysplipidemia in experimental animals [8]. Models in which rodents are fed diets with an excess of calories or in which rodents become obese because of genetic deficiencies or resistance to a satiety factor have been developed for NAFLD and atherosclerosis research [9-11]. High-frequency ultrasound has proven to be a useful tool for monitoring and assessing disease progression and pharmacological effects in small animals [12].

Ultrasound is an essentially subjective method for the diagnosis of liver diseases and carotid atherosclerosis, and its validity as an imaging method for evaluation of the liver has been described extensively for humans [6,13]. Several studies have also used ultrasonography to evaluate liver diseases in rodents [14,15]. Lessa et al. [16] and Lee et al. [17] compared histopathological findings to ultrasonographic B-mode quantitative findings in CCl_4_- and ethanol-induced fatty liver disease and cirrhosis. Matsuhashi et al. [18] and Layer et al. [19] correlated histology with analyses of the speed and texture of ultrasound in the liver, respectively. In addition, several studies have used ultrasonography to evaluate atherosclerosis in rodents [20,21], but there are few reports on carotid IMT in rodents. Studies comparing carotid IMT among rats with steatosis and steatohepatitis are lacking. The relationship between fatty liver diseases and carotid atherosclerosis in rodents is incompletely understood. There is a paucity of data regarding the correlation between carotid IMT and changes in hepatic pathology in rats with NAFLD.

The purpose of the present study was to determine the reliability of findings using high-frequency ultrasonic diagnostic equipment in the assessment of a rodent model of hepatic disease and early atherosclerosis induced by a high-fat diet in comparison with histological results. We attempted to assess carotid IMT in the follow-up in high-fat diet-induced NAFLD rats and ascertain if the degree of hepatic pathological changes was associated with carotid IMT.

We concluded that ultrasound imaging in the diagnosis of fatty liver disease and early atherosclerosis in rats is feasible and efficient, and that carotid IMT increased significantly in NASH rats but not in steatotic rats. A significant correlation between the IMT of the carotid arteries with hepatic inflammation score were shown in NASH rats. This method for non-invasive diagnosis is especially relevant in the research of the pathogenesis and therapy of NAFLD and atherosclerosis using rodent models or genetically deficient models.

## Results

### Hepatic ultrasound images in rats with NAFLD induced by a high-fat diet

**Figure 1:**
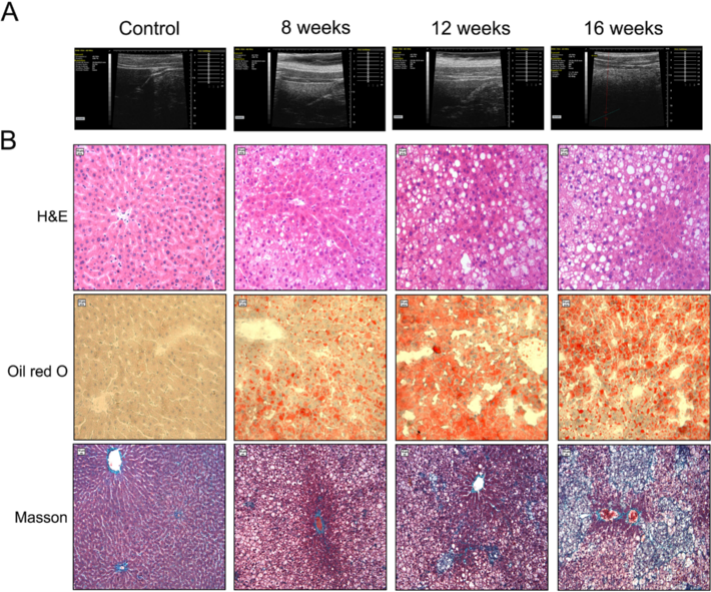
**Ultrasound and histological findings of livers from NAFLD rats fed a high-fat diet for 8, 12 and 16 weeks, respectively. (A)** Ultrasonographic representation (40 MHz) of the liver in transverse section. Transversal sonogram of Control group (normal liver) demonstrates homogeneous liver parenchyma, with medium level echogenicity and a regular hepatic surface (arrowheads). Transversal sonogram of liver in rats fed a high-fat diet for 8, 12 and 16 weeks, presents diffusely increased parenchymal echogenicity, the edge blunter, discrete coarse and heterogeneous parenchymal echogenicity and the liver surface as a dotted or slightly irregular line. **(B)** Histochemical analyses of liver specimens from rats stained with haematoxylin and eosin (H & E),oil red O and Masson’s. Rats fed a high-fat diet for 8 weeks show steatosis, predominantly as microvesicular fat in the acinar zones 1 and 2. Rats fed a high-fat diet for 12 and 16 weeks show pronounced hepatic steatosis, ballooning degeneration, infiltration of inflammatory cells (arrowhead), piecemeal necrosis (stars), fibrous tissue deposition and formation of fat granulomas (arrows). Objective lens, ×20.

All animals were examined by ultrasound before the rats were killed on the 8th, 12th and 16th week of induction of a high-fat diet. Healthy rats showed homogeneous liver parenchyma, with medium-level echogenicity and a regular liver surface. Liver echogenicity was lower in comparison to the right renal cortex, a finding that contrasts with results seen in humans. Fatty liver disease in rats was characterized by homogeneous and diffusely increased echogenicity (bright liver) equal or greater to the right renal cortex, reduced visualization of the diaphragm and of small peripheral vessels, and by no changes in the liver surface (Figure 1A), as described in humans [19,22]. The increase in liver echogenicity compared with the echogenicity in the right renal cortex had a sensitivity of 90% (24 out of 27) for the detection of fatty liver disease. Parameters such as an increased anteroposterior diameter of the liver and blunt edge of the liver were represented by changes in liver echogenicity, suggesting that hepatomegaly was also a prominent feature in NAFLD rats.

Evaluation of portal hypertension is also important for assessment of the severity of liver diseases. In the high-fat diet groups for 8, 12 and 16 weeks, the portal vein became wider and tortuous during the development of hepatic disease when compared with the control group. We analyzed the diameter of the portal vein among rats from 8-, 12- and 16-week high-fat diet groups with rats from the control group. The results were 1.97 ± 0.40 mm, 2.16 ± 0.42 mm, 2.19 ± 0.26 mm and 1.85 ± 0.25 mm, respectively. Portal-vein diameter was significantly different when comparing rats from 12- and 16-week high-fat diet groups with control rats (*P* < 0.01), suggesting mild portal hypertension. We also compared the velocity and quantity of the portal vein among rats from 8-, 12- and 16-week high-fat diet groups with rats from the control group. There were no significant differences among the groups evaluated, suggesting that mild portal hypertension did not lead to prominent hemodynamic changes in NAFLD rats. We also compared the index of spleen volume among rats from 8-, 12- and 16-week high-fat diet groups with rats from the control group. There were no significant differences among the groups assessed, indicating that splenomegaly was not a prominent feature in NASH rats.

### Hepatic histological analyses in rats with NAFLD induced by a high-fat diet

**Figure 2:**
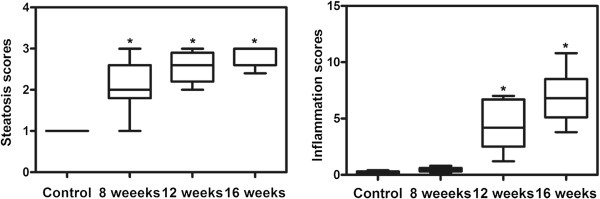
**The steatosis scores and the inflammation scores of the liver in NAFLD rats**. The steatosis scores and the inflammation scores were described in the “Methods” section. Data are medians (25/75th percentiles) for 9 rats per group. *P < 0.05 vs the control group.

Histology was used as the “gold standard” for the diagnosis of fatty liver disease and for the comparison with ultrasound results. The liver index of the high-fat groups was significantly more than that of the control group (*P < 0.01*), as described previously in rats [23], suggesting that hepatomegaly was a prominent feature in NAFLD rats. Rats in the control group showed a normal architecture with hepatocytes arranged in plates aligned to sinusoids converging to centrolobular veins. High-fat groups showed alterations in the liver parenchyma that correlated with the time of induction of injury. The prevalence of fatty liver disease among the 27 rats in the high-lipid groups was 100%. After 8 weeks of a high-fat diet (n = 9), half of the animals presented with mild steatosis, and the other half showed mild-to-moderate steatosis without fibrous septa. After 12 weeks and 16 weeks (n = 18), all animals showed moderate-to-marked steatosis. Apart from steatosis, ballooning degeneration, piecemeal necrosis, inflammation infiltrates, and early fibrosis were also observed in rats induced by a 12- and 16-week high-fat diet, respectively (Figure 1B). These changes were more prominent in the 16-week high-fat group. After 16 weeks, the animals showed minimal focal pericellular and perivenular fibrosis, as well as formation of fat granulomas even though liver parenchymal architecture was preserved, but no animals in the high-lipid groups presented with cirrhosis. H&E slides were analyzed using a semi-quantitative score for steatosis and inflammation, respectively. The degree of steatosis seen in the high-fat diet groups showed significant differences compared with the control groups (Figure 2A). The 12- and 16-week groups had a significantly higher inflammation scores than those of the control groups (Figure 2B). Microscopic evaluation showed indications of simple steatosis in rats induced by an 8-week high-fat diet. More importantly, histological analyses demonstrated that a high-fat diet for 12 weeks and 16 weeks could induce steatohepatitis (NASH) in rats (we refer to them as NASH rats).

### Hepatic lesions in rats with NASH induced by a high-fat diet

In comparison with the rats in the control group, serum levels of ALT, AST, TC, LDL and GLU increased in the high-fat diets groups as time progressed (Table 1). Moreover, these results were of greater severity at 12 and 16 weeks. Insulin levels and the HOMAIR index in the high-fat diet groups for 16 weeks were increased by about 60% and 90%, respectively, when compared with the control group. These data suggested insulin resistance in NASH rats induced by a 16-week high-fat diet.

**Table 1:** Biochemical changes in the blood

**Group**	**Control**	**8 weeks**	**12 weeks**	**16 weeks**
**ALT(U/L)**	**39.00 ± 7.05**	**52.44 ± 7.92**	**81.11 ± 31.28**	94.67 ± 44.38*
**AST(U/L)**	**99.44 ± 14.14**	**107.89 ± 36.92**	**154.33 ± 79.80**	206.56 ± 93.37*
**GLU(mmol/L)**	**5.89 ± 0.49**	**5.99 ± 0.58**	**6.01 ± 0.59**	6.9 ± 0.96*
**TG(mmol/L)**	**0.56 ± 0.12**	**0.57 ± 0.10**	**0.59 ± 0.13**	**0.61 ± 0.14**
**TC(mmol/L)**	**1.76 ± 0.28**	**1.97 ± 0.46**	**2.10 ± 0.48**	2.51 ± 0.59*
**HDL-C(mmol/L)**	**0.55 ± 0.10**	**0.53 ± 0.09**	**0.47 ± 0.10**	**0.50 ± 0.10**
**LDL-C(mmol/L)**	**0.24 ± 0.05**	0.68 ± 0.15*	0.83 ± 0.30*	1.21 ± 0.31*
**Insulin(**μ**IU/ml)**	**22.04 ± 7.46**	**25.93 ± 9.58**	**28.26 ± 9.45**	36.23 ± 9.28*
**HOMA**_**IR**_	**5.81 ± 2.04**	**6.89 ± 2.54**	**7.51 ± 2.29**	11.34 ± 4.17*

Data are mean ± SD, for 9 animals per group. *P < 0.05 *vs* Control. The control in table imply the control group (a standard diet) of 16 weeks. ALT, alanine aminotransferase; AST, aspartate aminotransferase; GLU, blood glucose; TG, triglyceride; TC, total cholesterol; HDL-C, high-density lipoprotein cholesterol; LDL-C, low-density lipoprotein cholesterol; HOMA_IR_, homeostasis model assessment insulin resistance index.

### Arterial lesions in rats with NASH induced by a high-fat diet

**Figure 3:**
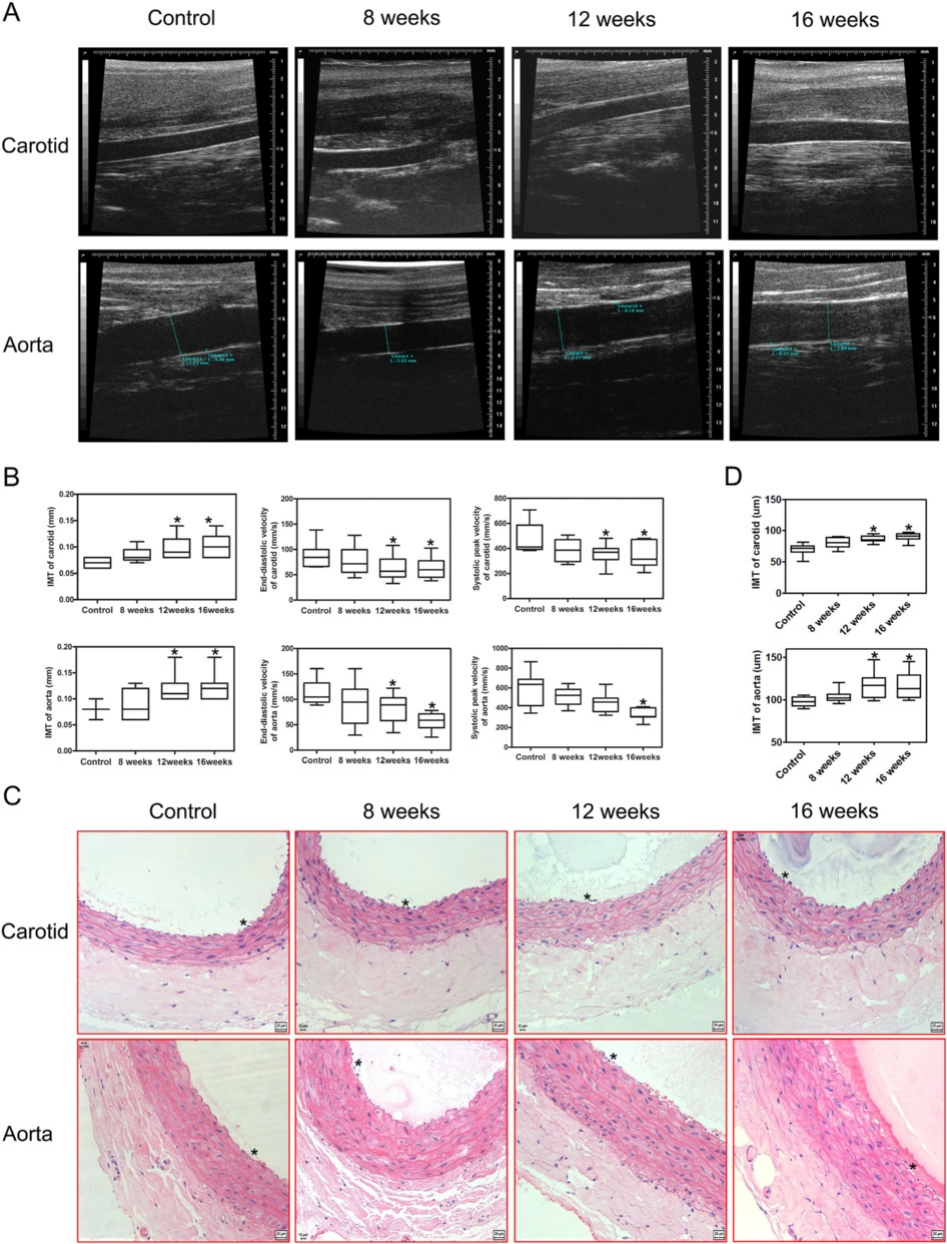
**Ultrasound and histological findings of the carotid artery and the aortas in rats. (A)** Ultrasound findings of the carotid artery and the aortas from rats fed a high-fat diet for 8, 12 and 16 weeks, respectively. **(B)** The IMT and hemodynamic parameters such as the end-diastolic velocity, the systolic peak velocity of the carotid artery and the aortas in rats fed with a high-fat diet for 8, 12 and 16 weeks,were measured by ultrasonography. **(C)** H&E staining of the carotid artery and the aortas sections (objective lens, ×20). The carotid artery and the aortas in rats fed with a high-fat diet for 12 and 16 weeks show mild intimal thickening and cellular hypertrophy in the intima and media of the carotid artery and the aortas. **(D)** IMT of the HE stained carotid artery and abdominal aorta was measured and quantified. Results represent medians (25/75th percentiles) of 9 rats per group. *P < 0.05 *vs*. the control group.

Among diagnostic imaging methods, ultrasound is considered to be ideal for the study of atherosclerosis [7]. To detect vascular lesions in rats with NAFLD induced by a high-fat diet, IMT and hemodynamic parameters was measured longitudinally in the carotid artery and aorta, respectively. IMT values for the carotid artery and abdominal aorta were remarkably different (P < 0.05) (Figures 3A and B). The lowest values were in the controls, intermediate values in rats at 8 weeks, and the highest levels in those with NASH (rats at 12 and 16 weeks). The diameters of the carotid artery and abdominal aorta in the NASH groups were smaller than those in the control group, but the difference was not significant (data not shown). We also assessed the hemodynamic parameters of the carotid artery and aorta among rats with steatosis and steatohepatitis. For rats at 8 weeks, there was a decreased trend in the end-diastolic velocity and systolic peak velocity of the carotid artery and aorta, but these values were not significantly different compared with those of the control group. The end-diastolic velocity and systolic peak velocity of the carotid artery and aorta in the NASH groups were significantly smaller than those in the control group (*P* < 0.05) (Figure 3B). These results suggested that early atherosclerosis may be present in NASH rats.H&E staining showed very mild intimal thickening, few foam cells in the intima and slight hyperplasia of the media of the carotid artery and aorta in the NASH groups. Plaques in the aortic intima were not observed by H&E staining or by macroscopic observation. This also demonstrated that vascular lesions were mild at the early stage of atherosclerosis (Figure 3C and D). The results of histopathological evaluation were consistent with ultrasonographic findings.

### Correlation between ultrasound findings and the histological diagnosis

**Figure 4:**
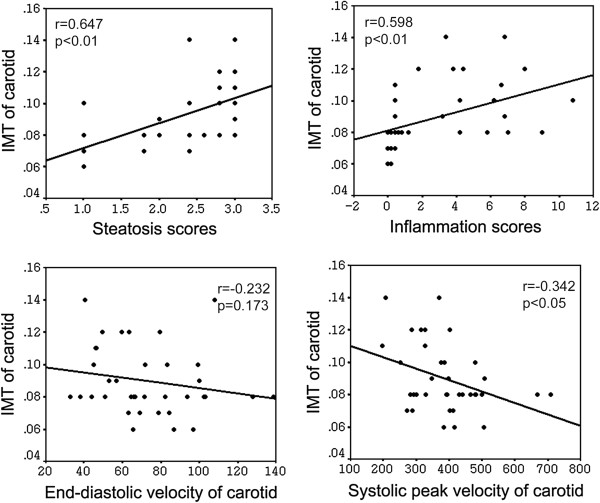
**Correlations between ultrasound analyses and the histological diagnosis.** Scatter plots of the IMT of the carotid artery and the steatosis scores of the liver, the inflammation scores of the liver, the end-diastolic velocity and the systolic peak velocity of the carotid artery in rats. Spearman rank test was performed to determine the correlations (r).

Figure 4 shows the parameters associated with carotid IMT in bivariate analyses. A significant correlation between the IMT of the carotid artery with hepatic steatosis score (*r*^*2*^ = 0.647, *P* = 0.000), hepatic inflammation score (*r*^*2*^ = 0.598, *P* = 0.001) and the systolic peak velocity of the carotid artery (*r*^*2*^ = −0.342, *P* = 0.041) were shown in the NASH groups, whereas the end-diastolic velocity of the carotid artery was not (*r*^*2*^ = −0.232, *P* = 0.173). These data suggested that steatosis and steatohepatitis were associated with carotid atherosclerosis.

## Discussion

NAFLD is the “hepatic expression” of the metabolic syndrome [4]. NAFLD patients are expected to have a higher risk of vascular disease and coronary heart disease because of the underlying metabolic disorder [5]. Follow-up mortality rates of NAFLD patients with coronary heart disease were found to be equal to those attributable to cirrhosis [5]. The severity of histopathological features in NAFLD is strongly associated with early carotid atherosclerosis, insulin resistance, and the metabolic syndrome [6,24].

Preclinical small-animal studies are a key part of medical and biological research. A rodent model of liver disease is the most commonly used model in preclinical studies [8]. We focused our attention in a rodent model of high lipid diet-induced NAFLD. We demonstrated that rats with access to a high-fat diet *ad libitum* for 8 weeks showed simple fatty-liver changes, and rats with access to this diet for 12 weeks and 16 weeks showed NASH changes (i.e. ballooning hepatocyte degeneration, inflammatory infiltrates, early fibrosis) in addition to steatosis along with other alterations in biochemical parameters. Especially in rats treated for 16 weeks, NASH severity was higher. This finding also illustrated that pathological changes in NASH rats was relatively long before the appearance of cirrhosis. Levels of AST, ALT, TC, LDL, GLU, and insulin as well as the HOMA_IR_ index increased remarkably in the 12- and 16-week groups. Thus, the histopathological and biochemical findings of the present study showed that a high-fat diet-induced model of NASH with insulin resistance in rats mimicked human NASH with respect to morphological and biological characteristics. This observation was in agreement with findings from studies of NASH patients [25] and an animal model of NASH induced by a high-fat diets [8]. More importantly, there was a significant correlation between carotid IMT and histology inflammation scores and steatosis scores in the livers of the NASH groups. To our knowledge, this is the first report of carotid IMT values among rats with NAFLD in longitudinal studies.

It is well known that ultrasound is essentially a subjective diagnostic method, and its validity for liver evaluation has been described extensively for humans [13,26], dogs [27] and cats [28,29]. High-frequency ultrasound has also been used in murine models of CVD [12,21]. However, dynamic changes in hepatic histology and carotid IMT in rats with NAFLD or atherosclerosis using ultrasound have not been reported. Our ultrasound results showed that fatty liver disease in rats was characterized by homogeneous and diffusely increased echogenicity (bright liver). Increases in liver echogenicity may be due to fatty infiltration and/or steatohepatitis, thereby changing the relationship between echogenicity in the liver and right renal cortex [13,30]. Our ultrasound findings were also comparable with histopathological changes in rats with access to a high-fat diet *ad libitum* for 8, 12 and 16 weeks. Portal hypertension is an important indicator of the severity of liver diseases and one of its most important signs is widening of the portal vein [16,31]. Portal-vein diameter was significantly higher in rats with steatohepatitis in comparison with the control group (Table 1), thereby suggesting mild portal hypertension. However, there were no significant differences in the velocity and quantity of the portal vein among the three groups assessed, suggesting that mild portal hypertension did not lead to prominent changes in hemodynamics in NAFLD rats.

Importantly, we demonstrated increases in the IMT values of the carotid artery and the aorta before plaque appearance using a high-frequency ultrasound method. Increased carotid IMT is a reflection of atherosclerotic burden and a predictor of subsequent events [7,32]. Measurement of carotid IMT is being employed increasingly frequently in clinical trials to assess the harmful effects of risk factors on vessel walls as well as the effect of treating risk factors that cause a reduction or prevent progression of IMT [7,20]. A change in the carotid IMT was a risk factor for atherosclerosis in patients with NAFLD, and the diagnosis of NAFLD was an independent predictor of increased IMT [5,6]. NAFLD patients showed greater carotid atherosclerosis with a higher mean IMT and higher prevalence of plaque formation. Hence, IMT is marker of early generalized atherosclerosis [7]. Comparison of carotid IMT using a rat model has not been carried out previously. Hence, we investigated if vascular injury occurred in high lipid diet-induced NAFLD rats. We demonstrated an increased IMT of the carotid artery and the aorta after administration of a high-fat diet for 12 weeks and 16 weeks. Functional and histopathological features in the carotid artery and aorta demonstrated changes in IMT values before the appearance of plaques. Assessment of the hemodynamic parameters of the carotid artery and the aorta among rats showed decreases in end-diastolic velocity and systolic peak velocity in the carotid artery and the aorta of rats in the 12 weeks and 16 weeks groups, respectively. This finding illustrated that vessel function had been reduced, including an increase in vascular resistance as well as a reduction in compliance and resilience in the NASH groups. These changes may be associated with an increase in arterial IMT in rats with NASH. We also demonstrated that a significant negative correlation between the carotid IMT with the systolic peak velocity of the carotid artery. It also demonstrated that functional disorders of vessel walls occurred in the early phase of atherosclerosis, and that rats with NASH had a greater prevalence of early atherosclerosis than controls. Histological change in arteries also supported a finding of early mild atherosclerosis. Interestingly, a significant positive correlation between ultrasonographic measurements in the carotid artery with the hepatic inflammation score and steatosis score was shown in the NASH groups, respectively. Hence, we demonstrated that NASH is a risk factor for early atherosclerosis in a rat model of NASH. This is the first report detailing percutaneous ultrasound of the carotid artery using a non-invasive, high-resolution system combined with an experimental model of NASH in rats to evaluate early atherosclerosis.

Usually, inducing atherosclerosis in normal rats and mice using a high-lipid diet is difficult because (i) rodents must be treated for a long time and (ii) complex traits for atherosclerosis are influenced by genetic variation and by the type of diet the rodents are fed. Hence, transgenic and mutant strains of mice with specific deficits or inducible atherosclerosis models are also often applied for atherosclerosis studies. So in this case, the development of a non-invasive modality for small-animal imaging is important for evaluating disease progression and pharmacological effects. Ultrasound is one of a useful method to longitudinal research on the same animal and to reduce requirements for animal sacrifice (e.g., studies for the metabolic syndrome, T2DM, atherosclerosis). We applied B-Mode (real-time imaging) and pulsed-wave Doppler mode to assess fatty livers, thickening of vascular walls and blood-flow velocity. We demonstrated that increased IMT and decreased hemodynamic functions of the carotid artery in response to high fat diet-induced NASH in rats could be used to visualize by employing a high-frequency ultrasound scanner. NAFLD models in rats are suitable to obtain ultrasound images, and can be applied to evaluate early atherosclerosis using IMT measurements. High-frequency ultrasound can be a powerful tool to monitor NAFLD and atherosclerosis progress in vivo, and could lead to longitudinal research on the same animal, shortened observation times, and reduced requirements for animal killing. Of course, further studies including the use of a higher-frequency ultrasound biomicroscopy or targeted molecular imaging technologies in assessment of real-time changes in vessel wall before the development of advanced atherosclerotic plaques for transgenic and mutant strains of rodents with specific deficits or inducible NASH are warranted to evaluate early pathophysiologic changes and reveal risk for future development of severe disease.

## Conclusion

We demonstrated that early atherosclerosis in NAFLD could be induced by a high-fat diet. Increases in the IMT values of the aorta and carotid artery in rats with NASH occurred before the appearance of plaques. The present study is the first to show that a significant correlation between the IMT of the carotid artery and hepatic inflammation score in rats with NASH can be induced by a high-fat diet.

## Methods

### Animals and diets

All animal protocols were approved by the Ethics Committee of the Capital University of Medical Sciences (Beijing, China). Animals were supplied by the Laboratory Animal Research Center of the same institution.

Fifty four male Sprague–Dawley rats (110–130 g) were used in the present study. After adaptation for 1 week, rats were divided randomly into two groups of 27. Animals in the control group had access to a standard diet *ad libitum*, and rats in the high-fat group had access to a high-fat diet [33]*ad libitum* for 8, 12 and 16 weeks, respectively. “the NASH groups rats” refer to rats in the high-fat diet for 12 weeks and 16 weeks, respectively. All animals were fed between 8 am and 9 am each day. They were maintained on a 12-h light/12-h dark cycle at 22–25°C and had access to sterilized water *ad libitum*. Body weight was recorded each week.

### Ultrasonography

Hepatic ultrasonography was conducted on all rats by two experienced examiners blinded to the study protocol using a Visual Sonics Vevo 770 Ultrahigh Frequency Ultrasound system. A transducer (Visual Sonics Vevo 770 plus RMV 704 or 710B) has a central frequency of 20–40 MHz providing an axial resolution of 40 μm with a 14.6-mm field of view and a centre focus at 6 mm. The scanhead operates with a frame rate of 32 Hz. The hemodynamics parameters of the vessel were detected using Doppler ultrasound. The organs and vessels in abdomen were scanned with transducer of RMV 704 or 710B, the image depth was about 12 mm, and the carotid artery was examined with transducer of RMV 704, the image depth was about 6 mm. The control and the high-fat groups were examined after a diet induction protocol for 8, 12 and 16 weeks, respectively. After an 10 h fast, rats were anesthetized using pentobarbital sodium. They were then placed on a platform in the supine position with the abdomen exposed to the transducer. The abdomen was shaved before imaging, and ultrasound gel applied gently to obtain better contact between the skin and transducer.

Animals were examined in the supine position to assess the liver, aorta, portal vein and the common carotid artery, and in the right posterior oblique position to assess the spleen. These organs were evaluated by multiple transversal and longitudinal scans. The acoustic focus was placed in the center of the target organ and in the largest transverse cross-section of the spleen. The portal vein was measured in the mid-point of the main portal vein using the calipers of the scanner. The brachydiagonal tangent plane of the common carotid artery was displayed by placing the probe beside the trachea. The liver plane was displayed by rotating the probe at 90°. We measured the carotid about at the 5 mm point proximal to the bifurcation of internal and external carotid artery. We measured the aorta at the 1 mm point proximal to the starting point of the renal artery. The diameter and IMT of arteries were measured [21,34]. Three values were measured on each side of the carotid artery, and the average IMT values used for the analysis. Seven consecutive cardiac beats were analyzed in two recorded times, and then the carotid diameter and carotid IMT obtained. The sample volume was put in the center of vessels, and the angle between the sound beam and blood flow was <60°. The images were analysed regarding IMT, first by one operator at two different ocarotid arterysions for validation of intraobserver variability, and then by a second operator for evaluation of interobserver variability. The measurement procedure should be demonstrated by a schematic diagram Additional file 1: Figure S1 and Additional file 2: Figure S2.

Ultrasonographic findings were analyzed based on criteria for ultrasound diagnosis in humans according to a classification based on three parameters. Firstly, hepatic steatosis was diagnosed and identified as an ultrasonographic pattern of a “bright liver”, with evident contrast between hepatic and renal parenchyma as described previously [16,22,35]. The pattern of of “bright liver” in two-dimensional ultrasound image is usually that of fine, closely packed echoes, and there is often hepatomegaly. ultrasound appearances of “bright liver” can be diagnosed by the observation of an increased hepatic echogenicity, characterized by hyperechoic liver parenchymal texture compared to the renal cortex, and decreased visualization of deeper structures, such as intra hepatic vascular vague. We set gray adjustable dynamic range of the instrument used in the same conditions to the exclusion of the fixing operation by the human factor. Secondly, the diameter of the portal vein was considered to be abnormal if ≥2.1 mm [16,35]. Thirdly, IMT was defined as the distance between the leading edge of the first echogenic line (lumen–intima interface) and the second echogenic line (media–adventitia interface) of the far wall. A carotid plaque was defined as a focal thickening ≥1.2 mm at the level of a carotid artery [32,36]. We measured the systolic peak velocity and end-diastolic velocity of the aorta and the carotid artery.

### Histopathology

After ultrasound imaging, rats were killed by puncture of the abdominal aorta. The livers, aortas and carotid arteries were removed rapidly and dissected for histological examination. Partial liver specimens and artery specimens were snap-frozen in liquid nitrogen and stored at −80°C for subsequent analyses. Bouin-fixed, paraffin-embedded sections of the liver and arteries were stained with hematoxylin & eosin (H&E) and Masson’s trichrome according to standard protocols. Sections of frozen rat-liver tissues were stained with Oil Red O for the detection of hepatic lipid droplets. A pathologist blinded to the animal grouping evaluated the slides and scored each liver tissue specimen based on the criteria proposed by Knodell, Lee and Brunt [25,37,38] for steatosis, inflammation and fibrosis. That is, for steatosis: grade 0, none present; grade 1, steatosis of <33% of the parenchyma; grade 2, steatosis of 34–66% of the parenchyma; grade 3, steatosis >67% of the parenchyma. For inflammation: grade 0, no foci of inflammation; grade 1, fewer than one foci per two 20 × fields; grade 2, one foci per two 20 × fields to one foci per one 20 × fields; grade 3, one to two foci per one 20 × fields; or grade 4, more than two foci per one 20 × fields. For fibrosis: grade 1, zone-3 perisinusoidal fibrosis; grade 2, zone-3 perisinusoidal fibrosis with portal fibrosis; grade 3, zone-3 perisinusoidal fibrosis and portal fibrosis with bridging fibrosis; and grade 4, cirrhosis. NASH was defined as steatosis plus lobular inflammation plus hepatocellular ballooning or steatosis plus any stage of fibrosis. IMT in HE stained carotid artery and abdominal aorta was measured by Pannoramic Viewer image analysis software (3DHISTECH, Ltd) following manufacturer instructions. Six values were obtained for each vessel and the average IMT values were used for the quantitative analysis.

### Serum measurements of biochemical markers

To measure blood glucose levels, serum activities of the liver-associated enzymes and serum lipids, artery blood samples were collected after 10 hours of fasting. Blood samples were centrifuged for 10 min at 500 g and at 4°C within 30–45 min of collection. Serum activities of the liver-associated enzymes alanine aminotransferase (ALT), aspartate aminotransferase (AST), as well as levels of blood glucose (GLU), triglyceride (TG), total cholesterol (TC), low-density lipoprotein cholesterol (LDL-C) and high-density lipoprotein cholesterol (HDL-C) were measured using commercial kits by an autoanalyzer in the Clinical Chemistry Laboratory of You An Hospital, Capital University of Medical Sciences. Blood glucose was determined by the enzymatic colorimetric method using glucose oxidase. ALT and AST were measured calorimetrically using standard kits purchased from Zhong Sheng Bei Kong Biolological Technology Co. Ltd (China). TG, TC, HDL-C and LDL-C levels were also measured by commercially available enzymatic reagents (Zhong Sheng Bei Kong Biolological Technology Co. Ltd.) adapted to the autoanalyzer. Serum levels of insulin were measured using a radioimmunoassay kit in the radioimmunological laboratory of hospital 301 (Beijing, China). Insulin resistance was calculated by means of the homeostatic model assessment index (HOMAIR) [39].

### Statistical analyses

Data are the mean ± SD or median and interquartile range, as appropriate. Skewed variables were transformed logarithmically to improve normality before analyses. Comparisons between groups were carried out using the ANOVA or Mann–Whitney U tests. Correlation among data was calculated by Pearson’s or Spearman’s test for non-parametric variables. *P* < 0.05 was considered significant. Statistical tests were conducted using SPSS ver15.0.

## Competing interests

The authors declare that they have no competing interests.

## Authors’ contributions

YJ and JW designed the research; YJ, JW, HZ and HZ collected the primary data; YJ and JW wrote the manuscript. All authors read and approved the final manuscript.

## Authors’ information

Jing Wu is first author.

## Supplementary Material

Additional file 1: Figure S1 A schematic diagram of the Doppler ultrasonic. The sampling frame is placed in the center of artery. D1 is the inner diameter of vessel. D2 is the internal-medial thickness of vessel. Q is the angle between the direction of blood flow and emitted sound. Click here for file

Additional file 2: Figure S2 A. Artery blood flow frequency spectrum. B. Venous blood flow frequency spectrum. Click here for file
