# Supplementary figures and images for: Hepatic inflammation scores correlate with common carotid intima-media thickness in rats with NAFLD induced by a high-fat diet
Source: BMC Vet Res. 2014 Jul 16;10:162. doi: 10.1186/1746-6148-10-162 (PMC4223401; doi:10.1186/1746-6148-10-162)

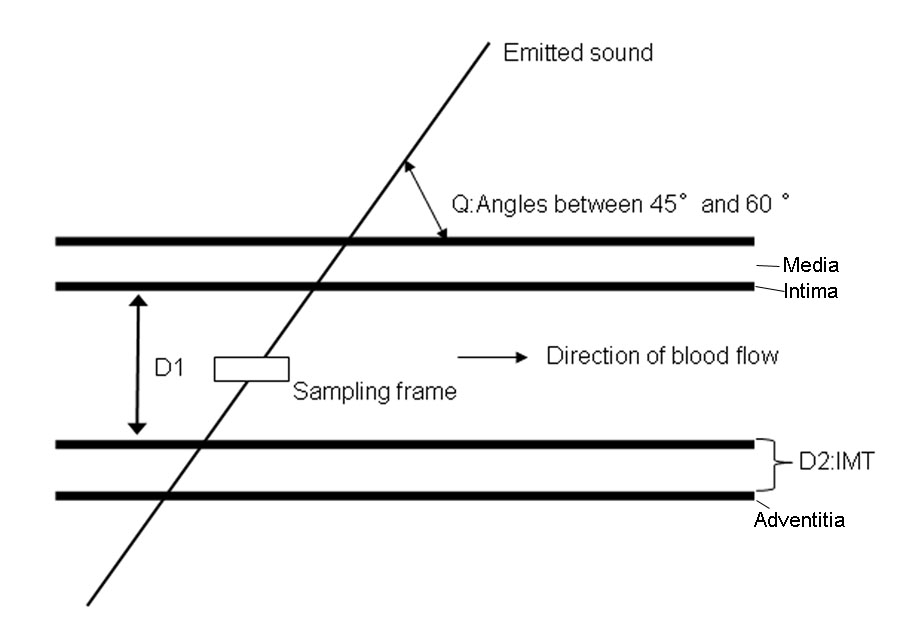

Supplement: Additional file 1: Figure S1 — A schematic diagram of the Doppler ultrasonic. The sampling frame is placed in the center of artery. D1 is the inner diameter of vessel. D2 is the internal-medial thickness of vessel. Q is the angle between the direction of blood flow and emitted sound. [file 1746-6148-10-162-S1.jpeg]

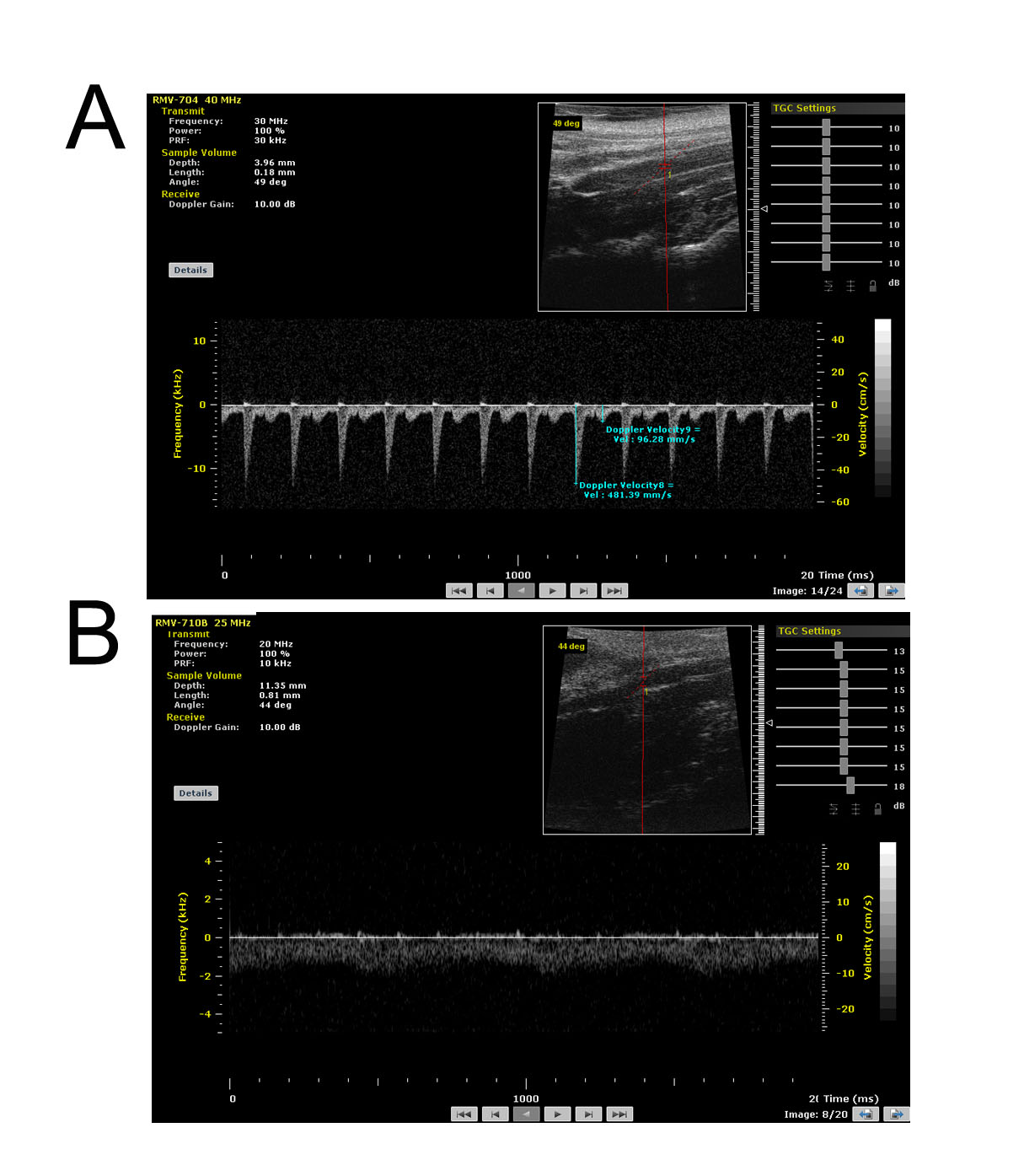

Supplement: Additional file 2: Figure S2 — A. Artery blood flow frequency spectrum. B. Venous blood flow frequency spectrum. [file 1746-6148-10-162-S2.jpeg]
